# Impacts of Solar Energy Development On Breeding Birds in Desert Grasslands In South Central New Mexico

**DOI:** 10.1007/s00267-024-02072-3

**Published:** 2024-12-10

**Authors:** Aaron C. Young, DeeAnne Meliopoulos, Martha J. Desmond, David Daniel, Fitsum Abadi

**Affiliations:** 1 Department of Fish, Wildlife and Conservation Ecology, New Mexico State University, Las Cruces, NM 88003 USA; 2 Department of Economics, Applied Statistics, and International Business, New Mexico State University, Las Cruces, NM 88003 USA

**Keywords:** Chihuahuan grasslands, Community occupancy, Grassland birds, Insectivorous species, Occupancy models, Synanthropic species

## Abstract

Solar energy is growing at unprecedented rates, with the most development projected to occur in areas with high concentrations of threatened and endangered species, yet its effects on wildlife remain largely unexplored. In 2014 and 2015 we examined the influence of a solar facility on avian community occupancy in the Nutt grasslands of south-central New Mexico. We examined the effect of distance to solar facility as well as other habitat covariates, including vegetation structure and orthopteran abundance, on community occupancy and occupancy trends for individual species. We did not find a significant effect of distance to solar facility on occupancy probability for the songbird community. Instead, orthopteran abundance had a significant positive effect on occupancy probability for the community. Two synanthropic species, Eurasian-collared dove (*Streptopelia decaocto*), and house finch (*Haemorhous mexicanus*), were found almost exclusively within the solar facility and both species increased between years, suggesting that developments in natural habitats may facilitate populations of synanthropic species. These results demonstrate the variability in responses of different species to a solar facility and the interacting influence of habitat characteristics and disturbance associated with development.

## Introduction

Solar energy is among the most rapidly expanding renewable energy forms (Lovich and Ennen, 2011; Walston et al., 2016). In the face of a changing climate, renewable energy sources have the potential to be major contributors to the reduction of carbon dioxide emissions (Sims, 2004; Tsoutsos et al., 2005). In comparison to conventional energy sources, solar energy has substantial environmental benefits, however, technical challenges related to capturing and storing energy still persist (Lewis and Nocera, 2006; Olabi and Abdelkareem, 2022). Despite the benefits of this green energy source for reducing greenhouse gas emissions, impacts to wildlife including conversion of habitat associated with facility operations remain under-explored. Therefore, solutions for placing facilities in landscapes with less potential for conflict with wildlife need to be investigated (Tsoutsos et al., 2005; Evans et al., 2023). Environmental issues exclusive to solar developments, specifically impacts on native communities, have only recently begun to be explored (Lovich and Ennen, 2011; Tawalbeh et al., 2021). To date, studies of the effect of solar development on wildlife have focused largely on avian mortality rates associated with collisions with panels or immolation due to flying into concentrated beams of sunlight (Walston et al., 2016; Conkling et al., 2022, Smallwood, 2022). The area in the United States currently occupied by solar energy development is expanding rapidly, estimated to be between 42,000 and 186,000 ha currently but projected to increase to 1,100,000 ha by 2030 (US Department of Energy, 2012; Solar Energy Industries Association, 2015). For example, in 2024 the Bureau of Land Management (BLM) has proposed to open 8,900,000 ha to solar energy development across 11 western states (BLM, 2024). A study in California found that most large-scale solar installations are sited in natural environments and near protected areas (Hernandez et al., 2015), suggesting a reduction in available habitat and further fragmentation of existing habitat. The region of maximum solar energy potential in the United States is restricted to six states in the southwest (US Department of Energy, 2012; US Department of the Interior, Walston et al., 2016). Development of solar facilities in this region is of particular concern due to its status as a “hotspot” for threatened and endangered species (Flather et al., 1998) and underscores the need to understand the effects of solar development on wildlife.

Many studies have examined effects of habitat fragmentation related to energy development on grassland birds, though the focus has most often been wind energy. Wind turbines in Texas displaced LeConte’s sparrows (*Ammodramus leconteii*) up to 400 m, and oil wells in Canada displaced Baird’s sparrows (*A. bairdii*) and Sprague’s pipits (*Anthus spragueii*) up to 450 m (Linnen, 2008; Stevens et al., 2013). Displacement may also occur through avoidance of edges produced by roads associated with development (Ingelfinger and Anderson, 2004; Dale et al., 2009; Carlin and Chalfoun, 2021). Vertical structures within open habitats are well established as a factor that can lower habitat use for grassland and shrubland songbirds, potentially due to increases in perceived or actual predation risk (Tack et al., 2017; Nenninger and Koper, 2018). Therefore, examining the potential effects of solar facility infrastructure on habitat use within the songbird community is a critical first step to understanding the ecological impacts of solar facility development.

Infrastructure and management activities associated with solar facilities may indirectly affect habitat use for the songbird community through altered vegetation structure (Conkling et al., 2022). Solar facilities may alter habitat vegetation to a greater or lesser degree, either by the complete removal of vegetation under solar panels (hereafter ‘blading’) or by reducing the height and/or cover of vegetation through management including mowing or herbicide. The majority of songbirds in Southwestern arid habitats are ground or shrub nesting species, and species associated with these habitats are not homogenous in their breeding habitat preferences (Fisher and Davis, 2010; Sadoti et al., 2018). Therefore, changes to vegetation characteristics associated with the establishment and operation of solar facilities are likely to affect individual species within the community in a manner dependent on the degree and type of vegetation change, given varied habitat associations. For species such as horned lark (*Eremophila alpestris*), which is associated with sparsely vegetated habitats, reductions in vegetation cover associated with energy facility maintenance may not impact habitat use (Beason, 2020). However, at facilities where blading occurs, habitat condition is unlikely to support ground-nesting songbirds regardless of habitat associations because all of the vegetation is removed. Vegetation structure may also impact the abundance and composition of the arthropod community, an important breeding season food resource for songbirds (e.g., Jirinec et al., 2016). Therefore, quantifying the potential effects of solar facilities on the distribution of preferred food resources for songbirds may be important for understanding the overall effect of energy facilities on habitat quality.

Many grassland birds become primarily or exclusively insectivorous during the breeding season (Wiens and Rotenberry, 1979), and changes to vegetative characteristics may alter the abundance or availability of insect prey. For example, native arthropod abundance has been found to be lower near edges and with decreasing fragment size in rangelands and grasslands (Ingham and Samways, 1996; Quinn, 2004; Prieto-Benitez and Mendez, 2011). However, solar facility operation does not necessarily preclude the maintenance of high arthropod diversity when native vegetation is retained or prioritized. In South Africa, Jeal et al. (2019) found no significant difference in arthropod abundances between a solar facility and adjacent rangeland, though diversity was higher in the rangeland and abundance of flying insects was higher in the solar facility, a factor that may influence songbirds that are aerial insectivores. Several U.S. states in the Midwest and East have enacted initiatives for solar energy development that prioritize the maintenance of native vegetation aimed at supporting populations of insect pollinators (Terry et al., 2020; Dolezal et al., 2021). However, research is lacking on the effects of vegetation alternation within solar facilities in the Southwest on the arthropod and bird communities, limiting the ability of developers to mitigate impacts on songbird populations.

In addition to the presence of physical infrastructure and potential changes to vegetative characteristics, solar facilities may alter microclimates through shading associated with raised solar panels. Evidence suggests that shade provided by solar arrays can decrease ground temperatures and increase soil moisture, important considerations for facilities in arid environments (Armstrong et al., 2016; Hassanpour Adeh et al., 2018). As the prevalence of drought and higher daily temperatures increase due to climate change, cooler microclimates associated with solar facilities may benefit ground-nesting birds by reducing heat stress and water loss (Smith et al., 2017; Ruth et al., 2020). Solar facilities in dryland settings may promote the recovery of native vegetation when management practices do not include blading and other disturbance activities (Xia et al., 2023). Further, management practices that promote higher diversity of native vegetation support higher arthropod diversity, a potentially important habitat characteristic for breeding songbirds (Blaydes et al., 2021).

Grassland and shrublands are the predominant habitats of the southwestern states designated for solar development, and over 99% of development is expected to occur in these ecosystems (Copeland et al., 2011; Hernandez et al., 2015). Grasslands are of particular concern as they have already undergone conversion to other habitat types more than any other North American biome (Sampson and Knopf, 1994; Noss et al., 1995; Brennan and Kuvlesky, 2005), and partly because of this grassland bird populations have experienced major declines (Ziolkowski et al., 2023). The Chihuahuan desert grasslands of south-central New Mexico are among the few remaining large expanses of desert grassland (Desmond and Montoya, 2006). However, these grasslands have undergone widescale changes due to increased shrub density and decreased grass cover associated with climate change and historic overgrazing (Yanoff and Muldavin, 2008, Christensen et al., 2023). In 2012, a 214-ha solar facility was established on the Nutt grasslands of central New Mexico. In this study, we modeled occupancy of avian species as a function of distance from the edge of the solar facility and incorporated the roles of vegetation and arthropod abundance. We predicted that: (1) community occupancy probabilities would differ on and off the facility with lower occupancy probabilities on the facility and higher off the facility; (2) Community composition would differ between plots on and off the solar facility due to loss of sensitive species on the facility and the addition of synanthropic species on the facility; (3) Biotic factors, including insect abundance and vegetation cover, could be more important than, or linked to, the direct effects of solar facility infrastructure on species-specific occupancy probabilities.

## Materials and Methods

### Study Area

The Nutt Grasslands are located in south-central New Mexico, in northeast Luna County and southwest Sierra County, in the northern part of the Chihuahuan Desert Ecoregion. In 2012, a 214-ha solar energy facility was constructed in the southwest part of the grasslands, 107°29'16.211“W, 32°34'18.87“N. This is a photovoltaic facility with a 55 MW capacity. It consists of 794,160 thin film photovoltaic solar modules, rated at 92.5 watts each, that are mounted on single-axis trackers (Southern Power, 2018). Solar arrays consisted of rows of 8–15 modules supported by metal beams. The height of the panels varied with site topography, but were approximately between 1.5 and 2.1 m. The solar facility had natural ground cover that was mowed but not bladed and dominated by native tobosa grass (*Pleuraphis mutica*). The boundary of the solar facility was marked by a chain-link fence surrounding all panels and related equipment. No pesticides or herbicides were sprayed within the facility during the course of this study. Our study area had an elevation of 1430–1485 m and annual precipitation averages of 24.54 cm (New Mexico Land Conservancy, 2011). During this study, precipitation was 5.01 cm below average in 2014, and 6.78 cm above average in 2015 (National Oceanic and Atmospheric Administration, 2016). Temperatures during the breeding season span an average low of 4.78 °C and an average high of 34.89 °C (New Mexico Land Conservancy, 2011). Chihuahuan Desert grasslands are dominated by black grama (*Bouteloua eriopoda*), six-weeks grama (*Bouteloua barbata*), and tobosa, with dominant shrub species being creosote bush (*Larrea tridentata*), honey mesquite (*Prosopis glandulosa*), and soaptree yucca (*Yucca elata;* New Mexico Land Conservancy, 2011). In addition to the solar energy facility, a 769-ha wind farm was installed immediately to the west of the solar development in 2011.

### Plot Establishment

**Fig. 1:**
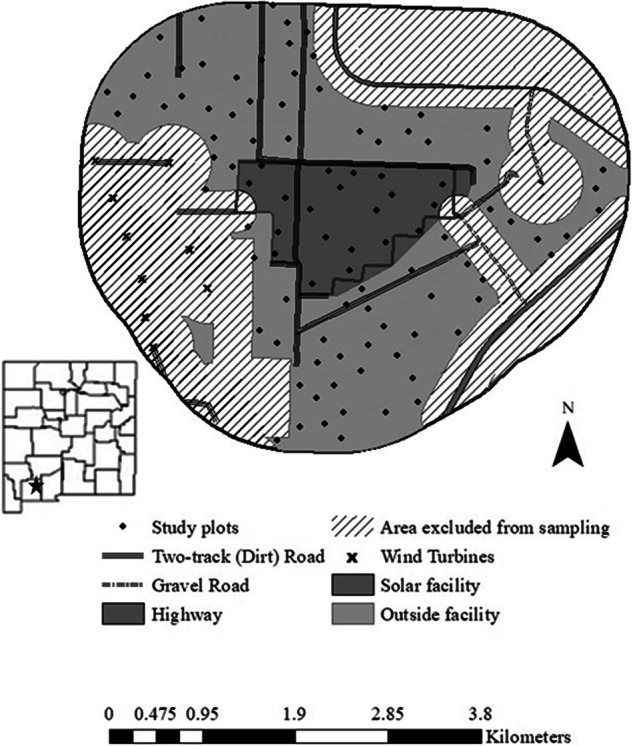
Study area and study plots for investigating effects of the Macho Springs Solar Facility on avian community occupancy in south-central New Mexico, 2014–2015

Our study design consisted of 100 plots, with 20 plots randomly placed within the solar facility and an additional 80 plots at stratified random locations up to 1600 m from the facility edge (Fig. 1). We placed plots outside of the facility within one of four distance categories (0–400 m, 401–800 m, 801–1200 m, and 1201–1600 m). We randomly placed 20 plots within each distance category. Each survey plot consisted of a single 50-m radius circle (Linnen, 2008). All plots were ≥200 m apart to prevent double-counting of territorial birds during surveys and ≥200 m from roads or other edges to minimize confounding edge effects and isolate effects of the solar facility (Best et al., 2001; Salek et al., 2010; Villegas-Patraca et al., 2012). In addition, all plots were >400 m from wind turbines, since birds have been found to avoid turbines within this distance (Stevens et al., 2013). The closest plot within the study area to a wind turbine was 438 m, and only three plots were between 400 and 500 m; most plots were greater than 1000 m from wind turbines. We created a GIS database to randomly select plot circle centers and checked points for homogeneity in vegetation and topography to minimize variation in species composition from differences in plot characteristics (Gilbert and Chalfoun, 2011).

### Avian, Invertebrate, and Vegetation Surveys

#### Avian Surveys

We conducted point count surveys for all species present in the community four times between April 15 and August 15 in 2014 and 2015. Point counts began at sunrise and continued for four hrs (Vickery et al., 1994; Linnen, 2008), in the absence of precipitation and with winds <20 km h^−1^ (Davis, 2004; Linnen, 2008). Each point count lasted five minutes, and any bird heard or seen within the 50 m radius from the center was recorded (Vickery et al., 1994; Ingelfinger and Anderson, 2004). Due to low abundance for multiple species in the community, we converted abundance data to presence/absence data for each of our four visits for each survey location.

#### Invertebrate Surveys

We sampled the insect community three times using pitfall traps between 4 May and 20 of August each year. Within each plot, we randomly located two pitfall traps, one within 25 m of the point center and the other within the outer 25 m (Niemela et al., 2002; Brust et al., 2005). All traps (*n* = 200) were sampled monthly for 7 straight days. We sorted and identified samples at the New Mexico State University (NMSU) entomology lab. Based on available literature we determined that crickets and grasshoppers (order Orthoptera), all beetles except darkling beetles (non-Tenebrionidae beetles) and spiders (order Araneae) were important in the diets of our dominant grassland birds (Wiens, 1973; Creighton and Baldwin, 1974; Wiens and Rotenberry, 1979; Linn, 2004).

#### Vegetation Sampling

Vegetative characteristics were measured each August using the line-intercept method (Herrick et al., 2009). We randomly placed four 25 m transects in each plot and averaged values for vegetation data over the four transects to yield one vegetation value for each plot for each year. We collected data at 1 m intervals along each transect on species composition, foliar coverage, and bare ground. We took visual obstruction readings at the start and end of each transect, using a Robel pole, for a total of eight visual obstruction readings (VOR) per plot (Robel et al. 1970). We averaged values for vegetation data over the four transects to yield one vegetation value for each plot.

#### Soil Temperature

We used iButtons (model DS1921G-F5#) in 2015 to log soil temperatures across plots for a comparison of temperatures among plots on the solar facility and at varying distances from the facility. We buried iButtons 2.54 cm below the soil surface (Anderson and Levine, 1987) at the plot center, at 10–18 plots per sampling period (mean = 14.5 plots per period). We compared iButtons on the solar facility (two plots) with iButtons off the facility (across two plots for each distance category) at two different times (1000 and 2000) on 6 May, 25 May, and 4 July, for a total of six comparisons and 8 temperature readings per comparison (Hu and Feng, 2003).

### Statistical Analysis

#### Comparison of Habitat Characteristics

We conducted preliminary analyses of differences in habitat characteristics within the solar facility and outside the facility. We compared soil temperatures between plots on and off the solar facility using a Kruskal-Wallis test. Six individual comparisons (*n* = 8 plots/comparison) were conducted (6 May, 25 May, and 4 July at 1000 and 2000 h). For each comparison, all soil temperature readings were taken within 5 min of each other. We compared habitat characteristics between locations within the solar facility and those outside the facility, including grass cover, forb cover, and orthopteran abundance, using linear regressions with a categorical predictor that indicated status as either inside or outside the facility.

#### Multiyear, Community Occupancy Models

To assess the effect of habitat characteristics and the solar facility on the songbird community, we first grouped observed species into 2 functional guilds: insectivores and synanthropic species. We defined insectivores in this instance as species that provide their nestlings invertebrates. We examined the effect of solar facility and habitat variables on community occupancy for insectivores and synanthropic species in 2014 and 2015 using a Bayesian multispecies dynamic occupancy model (Kéry and Royle, 2020). Multispecies inference has advantages over single-species modeling approaches in that more common species lend power to rare species resulting in improved parameter estimation when, as in our case, data for some species is sparse (Zipkin et al., 2010). We used the auto-logistic formulation of a dynamic occupancy model to place the focus of inference on occupancy probability as opposed to separate colonization and extinction parameters (Royle and Dorazio, 2008; Doser et al., 2022).

#### Model Specification

The formulation of the biological process model ${Z}_{i,k,t}$ is the true but unobservable occupancy status at site *i* of species *k* in year *t* which assumes a Bernoulli distribution with probability *φ*.

1 $${Z}_{i,k,t}\, \sim {Bernoulli}\left({\varphi }_{i,k,t}\right)$$

For the first year, we modeled the probability *φ* of species *k* occupying site *i* on the logit scale with a species-specific intercept (***β*0**) and species-specific regression coefficients (***β***) for the effects of site covariates. For example, a model that includes a single continuous covariate (${x}_{i}$) is

2 $${logit}\left({\varphi }_{i,k,1}\right)={\beta 0}_{k,1}+{\beta }_{k}* {x}_{i,1}$$

For the second year, our auto-logistic model formulation specified that the occupancy probability for species *k* was dependent on occupancy at site *i* the previous year, *t-1*, with the auto-logistic parameter (${\phi}_{k}$) for each species.

3 $${logit}\left({\varphi }_{i,k,t}\right)={\beta 0}_{k}+{\beta }_{k}* \,{x}_{i,2}+{{{\phi }}}_{k}* {z}_{i,k,t-1}$$

For the observation portion of our hierarchical model, ${y}_{i,j,k,t}$ represents detection/non-detection data and modeled as a Bernoulli random variable with parameter detection probability (${p}_{i,j,k,t}$) for species *k* at site *i* during replicate (visit) *j* in year *t* and the true occupancy status (${z}_{i,k,t}$).

4 $${y}_{i,j,k,t}\, \sim {Bernoulli}\left({p}_{i,j,k,t}* {z}_{i,k,t}\right)$$

We modeled detection probability as a function of covariates using a logit link function. With a single continuous covariate (${x}_{i,j,t}$), the model is given as

5 $${logit}\left({p}_{i,j,k,t}\right)=\,{\alpha 0}_{k,t}+\alpha * {x}_{i,j,t}$$

where ${\alpha 0}_{k,t}$ is species-and year-specific intercepts, and $\alpha$ is a fixed covariate effect.

Finally, for the insectivorous group we treated intercepts and covariate effects as random effects assumed to follow a normal distribution with a community level mean *μ* and variance *σ*^2^, which are shared by all *k* species.

6 $$\begin{array}{c}{\beta 0}_{k}\, \sim {Normal}({\mu }_{\beta 0},\,{{\sigma }^{2}}_{\beta 0})\\ {\beta }_{k}\, \sim {Normal}(\,{\mu }_{\beta },\,{{\sigma }^{2}}_{\beta })\\ {\alpha 0}_{k,t}\, \sim {Normal}({\mu }_{{\alpha }_{t,k}},\,{{\sigma }^{2}}_{{\alpha }_{t,k}})\end{array}$$

For the synanthropic group we treated intercepts and covariate effects as fixed effects because only 2 species were included in this group.

#### Model Selection

We included covariates in our model set that we a priori hypothesized would be important predictors of occupancy for the songbird community and that were indicated as influential by a preliminary analysis of this data (Meliopoulos, 2017). For our model set examining occupancy patterns for insectivorous species, we excluded mourning dove (*Zenaida macroura*) because this species is primarily granivorous throughout the year. For our model set examining occupancy of synanthropic species, we did not include arthropod abundance as a covariate in candidate models because neither synanthropic species we encountered primarily feed their nestlings arthropods (Badyaev et al. 2020, Romagosa and Mlodinow, 2022).

Our final model set included 12 models for the insectivorous group and 8 models for the synanthropic group, including a null model. We scaled and centered all continuous covariates and specified vague priors for hyperparameters (*μ* and *σ*). We selected the most parsimonious model by comparing deviance information criterion (DIC) scores (Spiegelhalter et al., 2002). We also assessed model fit using Bayesian *p*-values (Gelman and Shalizi, 2013). We conducted all analyses using JAGS called from program R version 3.0.3 (Plummer 2003, R Core Team, 2014).

## Results

Over the two years of this study, we completed 800 point-count surveys and detected 432 individual birds of 12 species. Six insectivore species had adequate sample size for analysis. Horned larks accounted for most detections in our raw data set (72.12%) followed by Chihuahua meadowlark (9.14%, *Sturnella lilianae*). Other species included in our analysis included Cassin’s sparrow (*Peucaea cassinii*), Western kingbird (*Tyrannus verticalis*), Say’s phoebe (*Sayornis saya*), and loggerhead shrike (*Lanius ludovicianus*). We encountered two species of synanthropic birds during the study, Eurasian-collared dove (*Streptopelia decaocto*) and house finch (*Haemorhous mexicanus*). House finches were not detected on plots in 2014 but were present in 2015 and, except for one pair 179 m from the facility, we observed this species solely on the facility. In both years, we detected Eurasian-collared doves exclusively on the facility (Table S1). Three waterbird species, great blue heron (*Ardea herodias*), spotted sandpiper (*Actitis macularius*), and white-faced ibis (*Plegadis chihi*) were observed inside the facility. These species were not included in our analysis.

### Habitat Characteristics

**Fig. 2:**
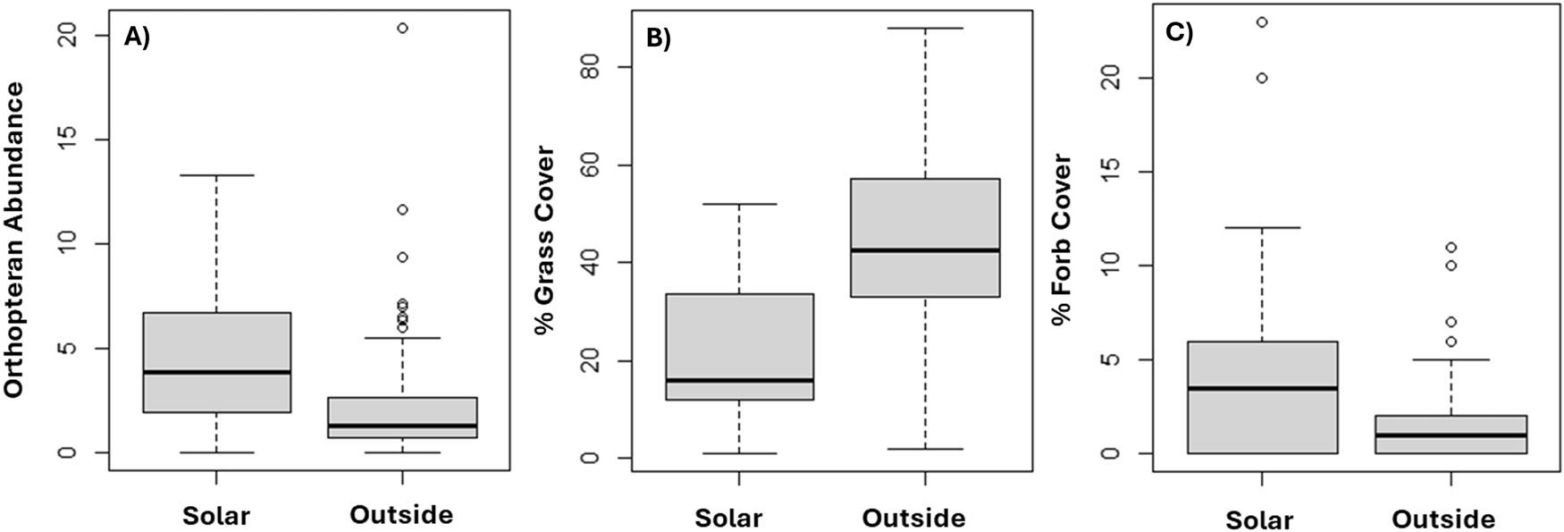
Box plots comparing habitat characteristics: **A** mean orthopteran abundance, **B** percent grass cover, and **C** percent forb cover within and outside of a solar facility in south-central New Mexico, 2014–2015. Vegetation within the solar facility was mowed but not completely removed

Soil temperatures in 2015 were significantly lower on the solar facility, primarily in the afternoon, compared to plots off the facility in four of the six temperature datasets with average soil temperatures reported for paired comparisons on and off the solar facility (Table S2). Soil temperatures in the facility ranged from 1.76 to 5.47 °C lower than the surrounding undeveloped habitat. Preliminary data exploration of univariate arthropod models (Spider, Beetle, Orthopterans, and Total Insects) revealed that Orthopteran abundance best predicted community occupancy probability (Table S3). Orthopteran abundance was significantly higher on survey locations inside the facility compared to those outside (β = −0.88, SE = 0.17, *p* = 7.7e-07; Table S4, Fig. 2A). Mean percent grass cover was significantly higher outside the facility compared to inside (β = 1.27, SE = 0.16, *p* = 1.39e-13, Table S4, Fig. 2B), while mean percent forb cover was higher inside the facility than outside (β = −1.00, SE = 0.17, *p* = 1.47e-08, Table S4, Fig. 2C).

### Insectivorous and Synanthropic Species

**Fig. 3:**
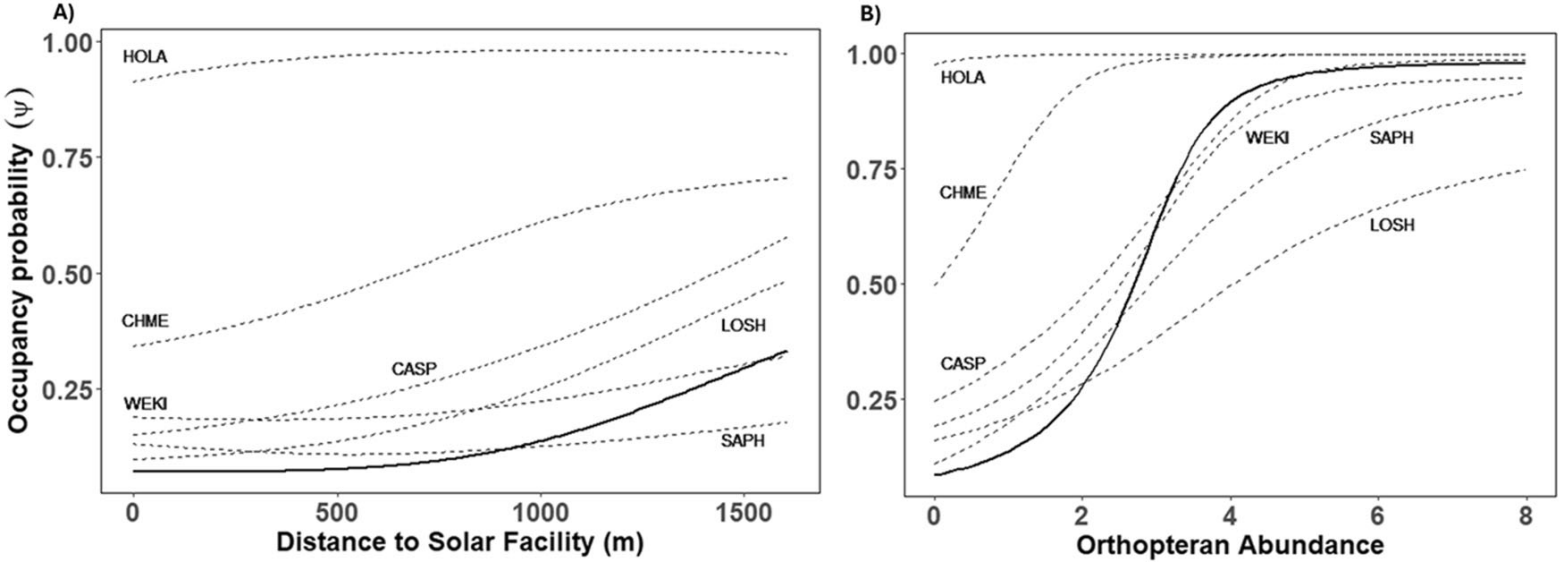
Predicted species-specific (dashed line) and population-averaged (community; solid line) occupancy probability from the most supported model in relation to **A** distance to solar facility on occupancy probability when orthopteran abundance is lowest; **B** orthopteran abundance while setting distance to solar facility at its mean value. Species represented include horned lark (HOLA), Chihuahuan meadowlark (CHME), Western Kingbird (WEKI), Cassin’s sparrow (CASP), loggerhead shrike (LOSH), and Say’s phoebe (SAPH)

**Fig. 4:**
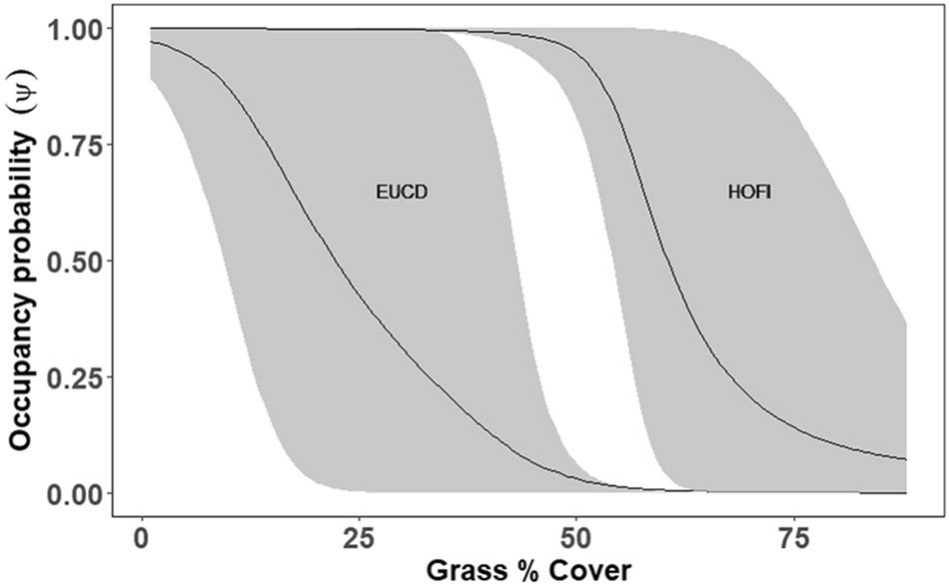
Predicted species-specific occupancy probability (solid line) and 85% credible intervals (shaded area) for synanthropic species from the most supported model in relation to % grass cover while setting forb cover at its mean value. Species represented include Eurasian-collared dove (EUCD) and house finch (HOFI)

A model including distance to solar farm, orthopteran abundance, and forb cover was the best supported model for community occupancy probability for insectivorous species (Table 1). Detection was low for all but the most common species (Table 2). The R-hat values and visual inspection of MCMC diagnostic plots indicated model convergence, and the Bayesian *p*-value for our top model suggested adequate model fit (*p*-value = 0.49). Though community occupancy probability increased as distance to solar facility increased, this trend is not statistically significant (β = 2.24, 85% credible interval (CrI) −1.53, 3.83), Fig. 3A. Instead, occupancy probability significantly increased in response to orthopteran abundance (β = 7.81 85% CrI 1.63, 14.21), Fig. 3B. Credible intervals for the estimated effect of forb cover on occupancy probability crossed zero (β = −1.45, 85% CrI −6.52, 3.72). For synanthropic species, a model that included grass cover and forb cover was the best-supported model (Table 1). Occupancy probabilities for both Eurasian-collared dove and house finch declined as grass cover increased, though credible intervals overlapped for house finch (EUCD β = −7.95, 85% CrI −14.27, −2.60, HOFI β = −5.66, 85% CrI −12.46, 1.43, Fig. 4). Occupancy probability for Eurasian-collared dove and house finch increased as forb cover increased (EUCD β = 4.47, 85% CrI −0.66, 9.89), HOFI (β = 5.13, 85% CrI −0.60, 11.92) but credible intervals for both marginally overlapped zero.

**Table 1:** Model selection results for occupancy probability of the autologistic dynamic occupancy models using the deviance information criterion (DIC) and Bayesian p value for evaluating model fit

Model	DIC	ΔDIC	Bayesian *p* value
Insectivorous Species			
Solar + Insect + Forb	1405	0	0.496
Solar + Insect	1454	49	0.498
Solar + Grass + Insect + Forb	1464	59	0.497
Solar + Insect + Grass	1582	177	0.493
Solar + Forb	1602	197	0.492
Solar + Grass + Forb	1641	236	0.496
Insect	1712	307	0.490
Solar + Grass	1761	356	0.495
Forb	1836	431	0.489
Grass	1855	450	0.488
Solar	1949	544	0.487
Null	2008	603	0.477
**Syanthropic Species**			
Forb + Grass	280	0	0.41
Solar + Forb + Grass	284	4	0.41
Solar + Grass	285	5	0.40
Solar + Forb	289	9	0.42
Grass	289	9	0.41
Solar	293	13	0.42
Null	293	13	0.42
Forb	301	21	0.42

Covariates in models included combinations of distance to the solar facility (Solar), orthopteran abundance (Insect), percent cover of forbs (Forb), and percent grass cover (Grass).

**Table 2:** Estimated regression coefficients (with 85% confidence interval in parentheses) from the most supported model for avian community response to solar facility

Species	Intercept	Distance to Solar Facility	Orthopteran Abundance	Forb Cover	Detection	
					2014	2015
HOLA	12.59 (4.23, 20.55)	1.02 (−2.30, 4.61)	3.61 (−2.71, 10.44)	0.39 (−3.62, 4.70)	−1.25 (−1.43, −1.06)	−0.83 (−1.00, −0.67)
CHME	9.03 (2.41, 15.77)	1.72 (−1.90, 6.45)	8.12 (0.61, 16.11)	−1.19 (−6.31, 4.44)	−3.92 (−4.51, 3.33)	−2.64 (−2.99, −2.31)
CAFI	2.87 (−5.26, 11.18)	4.32 (0.16, 9.13)	9.14 (1.62, 17.12)	−6.90 (−17.56, 2.17)	−4.88 (−5.99, −3.75)	−3.10 (−3.66, −2.55)
WEKI	1.90 (−5.19, 9.88)	1.45 (−4.66, 7.48)	9.69 (0.18, 19.79)	−4.21 (−14.98, 4.41)	−5.33 (−6.75, −3.93)	−4.46 (−6.37, −2.51)
SAPH	0.91 (−11.37, 11.85)	0.72 (−5.55, 6.05)	11.06 (2.28, 19.50)	0.05 (−5.46, 5.78)	−6.19 (−8.76, −3.86)	−3.35 (−4.90, −1.78)
LOSH	−2.10 (−14.15, 9.20)	4.29 (−1.52, 11.71)	6.83 (−1.39, 15.84)	1.52 (−6.46, 10.85)	−6.02 (−8.85, −3.50)	−4.47 (−5.87, −3.12)

The intercept (logit), covariate, and detection (logit) parameters are species-specific random slopes. *HOLA* Species represented include horned lark, *CHME* Chihuahuan meadowlark, *WEKI* Western Kingbird, *CASP* Cassin’s sparrow, *LOSH* Loggerhead shrike, and *SAPH* Say’s phoebe

## Discussion

The development of renewable energy is an increasingly important component of efforts to combat the impacts of climate change, and siting of solar facilities in the southwestern U.S. is increasing and is projected to grow substantially (Gibson et al., 2017; BLM, 2024). While the benefits of renewable energy development for climate mitigation are well understood, questions remain over the impacts of facilities on sensitive wildlife populations. For songbirds, multiple studies have examined the direct effects of solar and wind facilities on population demographics by quantifying mortality rates, caused by either collision with wind turbines or singeing caused by solar facilities (Kosciuch et al., 2020). Fewer studies have examined the indirect effects of solar facilities on the songbird community (Smith and Dwyer, 2016). Our study highlights how habitat characteristics that influence food resources can have a stronger effect on songbird community composition than any effects of solar facility structures.

Habitat associations of grassland birds vary by species within the community, as does the response of individual species to vertical structures within the habitat matrix. Species of grassland birds can be sensitive to vertical structures, for example by avoiding woody edges (Chapman et al., 2004; Thompson et al., 2014) or wind turbines (Coppes et al., 2020). Alternatively, grassland songbirds may use vertical structures as singing perches, a common behavior for species such as meadowlarks (*Sturnella spp*., Rodgers and Koper, 2017). Results from our study suggest that any negative response of the bird community in the Nutt grasslands to solar facility structures is mitigated by the abundance of food resources present within the facility. The most common species in our study are ground nesting species including horned lark, the most common species at the site, which is associated with bare ground and sparse vegetation. Therefore, the presence of spatially heterogeneous native vegetation that is periodically mowed may be beneficial for this species. Though we only found evidence of a positive association with the solar facility for synanthropic species, life histories and naïve occupancy patterns suggest the solar facility may provide resources otherwise absent in an arid grassland for other species as well. For example, Say’s phoebe, a species that hunts from perches and commonly nests on buildings and other human structures, was only present on the solar facility (Schukman and Wolf, 2020, Table S1).

Habitat alterations associated with solar facility development have the potential to alter ecosystem processes at lower trophic levels, thereby altering resource availability for songbirds. For example, mowing within a solar facility alters the floral and vegetative characteristics of the habitat which can impact the insect community by lowering the abundance of insect pollinator species (Walston et al., 2018; Grodsky et al., 2021). In contrast, shading by photovoltaic panels can delay timing of flowering for forbs, thereby increasing insect pollinator abundance later in the summer in dryland environments (Graham et al., 2021). Soil temperatures were substantially cooler on the facility; this appeared to be a combined result of shade from the solar panels reducing heat absorption by surface soils and increased cooling due to transpiration through the retention of natural vegetation on the solar facility floor (Smith et al., 1987; Barron-Gafford et al., 2016). Vegetation is often removed at solar facilities, creating a heat island effect where photovoltaic arrays are warmer during both day and night due to increased heat retention and lower evapotranspiration associated with reduced plant biomass (Barron-Gafford et al., 2016). However, in our study, the native vegetation (although kept low by mowing) was retained on the installation floor. The retention of native cover and resulting lower temperatures may have influenced orthopteran abundance, which was higher within the solar facility than outside the facility, a finding consistent with other research (Jeal et al. 2019). Orthopterans are a preferred food item for adult grassland birds in the breeding season, as well as an important component of nestling diets (Wiens and Rotenberry, 1979), and heterogeneous grass cover, i.e., sparse cover interspersed with un-grazed or un-mowed cover, can lead to increased abundances of orthopteran species (Gardiner, 2018). Reductions in grass cover associated with practices such as blading can also impact habitat microclimate, increasing soil temperature and reducing shaded cover, leading to lower orthopteran abundance (O’Neill et al., 2003; Gardiner and Hassall, 2009). The structure of vegetation on the solar facility, combined with an abundance of perches and lower ground temperatures associated with solar panels, may facilitate hunting of orthoptera by songbirds enough to outweigh other habitat-quality tradeoffs. In other studies, abundance of overwintering warblers was predicted by arthropod biomass rather than by vegetation features or predator abundance (Johnson and Sherry 2001), and lesser prairie-chickens (*Tympanuchus pallidicinctus*) selected for habitat with higher orthoptera biomass (Jamison et al., 2002). The positive response of individual species and the community as a whole to orthopteran abundance suggests that food resources are driving community distributions.

Development of infrastructure in wildland settings can alter wildlife community structure by providing resources to generalist species while reducing habitat-use for sensitive and/specialist species. As mentioned above, it is noteworthy that we detected synanthropic species almost exclusively on the solar facility. The sole detection of synanthropic species outside the facility was a single pair of house finches adjacent to the solar facility edge. House finches were not detected on plots on the solar facility until two years after construction of the facility (2015) and their habitation, along with a two-fold increase in relative abundance of Eurasian collared-doves between years, resulted in a more than eight-fold increase in synanthropic species between years. This increase was disproportionate to the increase in overall avian relative abundance between years, supporting other studies that have found that synanthropic species thrive and multiply over time in developed areas (Johnston, 2001; Pidgeon et al., 2014; Wood et al., 2015). DeVault et al. (2014) observed more house finches and European starlings (*Sturnus vulgaris*) on solar installations than in adjacent airfields, and in South Africa, Jeal (2017) documented house sparrows (*Passer domesticus*) on a solar installation but not on surrounding rangelands. Eurasian-collared dove abundance rose by a factor of almost seven over a two-year period in Florida, with adaptation ability cited as a principal cause of this increase (Romagosa and Labisky, 2000). In Mexico, Eurasian-collared doves where more strongly associated with human development than other dove species (Camacho-Cervantes and Schondube 2018) The positive influence of solar-facility development on habitat use by non-native and synanthropic species could represent a loss of scarce resources for native species, but further study is required.

Low avian diversity is a general characteristic of grassland ecosystems, including desert grasslands (Agudelo et al., 2008). The three most common species at our site, horned lark, mourning dove, and Chihuahuan meadowlark, are among the most commonly observed species at solar facilities in the southwest, considering both mortalities and live observations (Kosciuch et al., 2020; Conkling et al., 2022). It may be that these species are less likely to be affected by solar farm development, and that other more sensitive species have already been extirpated from the community. If this is the case, long-term before-after control-impact studies may provide a more complete inference for the effect of solar facility development on songbird community composition. Our use of a community occupancy model facilitated the inclusion of less abundant species in our analysis. However, we acknowledge that the use of an occupancy model as opposed to an abundance model represents a loss of information for more abundant species. Further, the use of a community model may mask reponses of rare species because covariate effects tend to shrink toward the mean of more common species.

The strongest observed response to the solar facility was the fairly rapid colonization of the solar facility by synanthropic species, likely from source populations on surrounding ranches. Other factors that may have contributed to a lower response to the facility’s presence include the relatively small size of the Macho Springs solar facility and a low diversity of breeding grassland birds in this region. Studies reporting strong effects of energy development on birds have been conducted on installations covering larger areas (Leddy et al., 1999; Dale et al., 2009; Gregory and Beck, 2014). Not surprisingly, the size of the development has been linked to the magnitude of impact (Dale et al., 2009). Strong negative effects on avian abundance from a concentrated solar power facility have been found in South Africa at a facility only 25% larger than the Macho Springs facility (Jeal, 2017). Further, many studies have documented lags in effects of energy development on bird populations because breeding site fidelity may buffer any large effect on community composition for several years (Stewart et al., 2007; Harju et al., 2010; Hess and Beck, 2012; Gregory and Beck, 2014). Some studies identify a minimum monitoring period of five years to fully realize effects of development on species abundance and community composition (Stewart et al., 2007; Gregory and Beck, 2014), suggesting that the length of our study may not have been sufficient to fully document effects. Additionally, a stronger response may have been observed during migration when a higher diversity of grassland bird species passes through this area (Agudelo et al., 2008). During this time, there might also be an increased response of water birds to the solar facility due to the “lake effect” (Kagan et al. 2014). A potential limitation of our data is the use of pitfall traps to estimate arthropod abundance, which may not adequately sample groups associated with upper vegetation layers (Cooper and Whitmore 1990). Our data therefore represents an estimate of relative abundances across the study site. The results of this study will provide a foundation for continued exploration of the effects of solar energy facilities on songbirds. Understanding the scale, both temporal and spatial, of any effects of human development as well as the direct and indirect effects of development on the community within the surrounding habitat matrix is vital for predicting the effect of renewable energy development on wildlife populations.

### Conclusions

Ideally, developers should site facilities in areas that are already disturbed to prevent fragmentation of intact habitat and retain native vegetation on the floor of the installation instead of blading the ground to prevent the heat island effect (Barron-Gafford et al., 2016). In the southwestern United States, this could include degraded sites that have been impacted by desertification (Herbal et al., 1972; Neilson, 1986; Schlesinger et al., 1990; Peters et al., 2015). Solar facilities should be monitored for longer time periods to document full effects. Studies should also monitor avian responses over different periods of the annual cycle. Although the breeding period is important and often easier to study, the migration and winter periods may present alternative or stronger results due to differences in avian community composition and associated levels of sensitivity. The spike in synanthropic species, an avian group observed only on or near the facility, may indicate challenges to preserving native communities, especially if population growth leads to spillover to the surrounding habitat. Perhaps most importantly, management of vegetation within and outside of the development that prioritizes floral and vegetative characteristics that support a diverse and abundant insect population may ameliorate potential negative effects of solar development for breeding songbirds.

## Supplementary information

Supplement

## Data Availability

Data will be provided contingent on publication of this manuscript at https://github.com/achristophery/SolarEnergyBreedingBirds.
