# Supplementary material for: Impacts of Solar Energy Development On Breeding Birds in Desert Grasslands In South Central New Mexico
Source: Environ Manage. 2024 Dec 10;75(4):883–95. doi: 10.1007/s00267-024-02072-3 (PMC11965265; doi:10.1007/s00267-024-02072-3)
Supplement: Supplementary file 1 — Supplement [file 267_2024_2072_MOESM1_ESM.docx]

**Supplement:** **Impacts of Solar Energy Development on Breeding Birds in Desert Grasslands in South Central New Mexico**

Journal of Environmental Management

Aaron C. Young^1^, DeeAnne Meliopoulos^1^, Martha J. Desmond^1*^, David Daniel^2^, and Fitsum Abadi^1^

1. Department of Fish, Wildlife and Conservation Ecology, New Mexico State University, Las Cruces, NM 88003, USA
2. Department of Economics, Applied Statistics, and International Business, New Mexico State University, Las Cruces, NM 88003, USA

*Corresponding author: [mdesmond@nmsu.edu](mailto:mdesmond@nmsu.edu), Department of Fish, Wildlife and Conservation Ecology, PO 30003, MSC 4901, Las Cruces, NM 88003-0003

**Table S1**. Relative abundance of individual species at a solar facility in south-central New Mexico in 2014-2015. Survey locations were either within the solar facility boundary (*n* = 20) or outside the facility boundary (*n* = 80). We generated relative abundance estimates using a generalized linear model with a categorical predictor for location as either within or outside the solar facility with 95% confidence intervals in parentheses.

| **Species** | | **Relative Abundance** | | | |
| --- | --- | --- | --- | --- | --- |
|  |  | **2014** | | **2015** | |
|  |  | **On-Facility** | **Off-Facility** | **On-Facility** | **Off-Facility** |
| Horned Lark | 1.52 (1.05, 2.11) | | 0.67 (0.50, 0.86) | 1.28 (0.85, 1.83) | 1.39 (1.14, 1.66) |
| Mourning Dove | 0.09 (0.01, 0.29) | | 0.10 (0.04, 0.19) | 0.43 (0.20, 0.77) | 0.12 (0.06, 0.22) |
| Meadowlark | 0.19 (0.05, 0.44) | | 0.07 (0.03, 0.15) | 0.14 (0.03, 0.37) | 0.26 (0.16, 0.39) |
| Cassin’s Sparrow | Absent | | 0.02 (0, 0.07) | 0.04 (0, 0.20) | 0.13 (0.07, 0.23) |
| Western Kingbird | Absent | | 0.02 (0, 0.07) | Absent | 0.01 (0, 0.05) |
| Say’s Phoebe | Absent | | Absent | 0.09 (0.01, 0.29) | Absent |
| Loggerhead Shrike | Absent | | Absent | 0.04 (0, 0.21) | 0.01 (0, 0.05) |
| Eurasian Collared-Dove | 0.09 (0.01, 0.29) | | Absent | 0.19 (0.05, 0.44) | Absent |
| House Finch | Absent | | Absent | 0.43 (0.20, 0.77) | 0.02 (0, 0.07) |

**Table S2.** Mean soil temperatures inside and outside of a solar energy facility in 2014 in south central New Mexico. We collected temperature readings (*n =* 48) on three days during the breeding season at 10 am and 8 pm.

| **Sample** | **In Facility** | **Outside Facility** | ***X^2^*** | ***P*** |
| --- | --- | --- | --- | --- |
| May 6, Morning | 21.50°C ± 1.50 | 23.94°C ± 0.68 | 2.485 | 0.110 |
| May 6, Afternoon | 22.00°C ± 0.50 | 26.08°C ± 0.29 | 7.030 | 0.027 |
| May 25, Morning | 27.50°C ± 0.50 | 29.26°C ± 0.93 | 2.793 | 0.090 |
| May 25 Afternoon | 23.25°C ± 0.25 | 26.67°C ± 0.54 | 3.882 | 0.050 |
| July 4, Morning | 25.89°C ± 1.37 | 28.11°C ± 0.22 | 3.799 | 0.050 |
| July 4, Afternoon | 25.09°C ± 0.37 | 30.56°C ± 0.40 | 5.798 | 0.020 |

**Table S3.** Model selection results for univariate models of the effect of arthropod abundance on community occupancy probability. Models were ranked using the deviance information criterion (DIC).

| **Arthropod Abundance Covariate** | **DIC Score** | **ΔDIC** |
| --- | --- | --- |
| Orthoptera | 1667 | 0 |
| Total Arthropod | 1765 | 98 |
| Spider | 1930 | 263 |
| Beetle | 2008 | 341 |

**Table S4.** Habitat characteristics within and outside a solar facility in south-central New Mexico in 2014-2015. Orthopteran abundance represents the mean relative counts at sampling locations. Grass cover and forb cover represent the mean percent covers at sampling locations. 95% quantiles are in parentheses.

| **Covariate** | **Within Solar Facility** | | **Outside Solar Facility** | |
| --- | --- | --- | --- | --- |
|  | **2014** | **2015** | **2014** | **2015** |
| Orthopteran abundance | 4.40 (1.33, 7.42) | 4.75 (1.66, 7.17) | 2.25 (0.33, 3.76) | 1.98 (0.50, 3.27) |
| Grass % cover | 12.27 (5.10, 20.45) | 30.44 (14.55, 44.45) | 50.28 (36.0, 65.0) | 39.35 (25.0, 56.15) |
| Forb % cover | 0.94 (0, 2.45) | 9.77 (4.0, 20.0) | 0.98 (0, 2.0) | 2.3 (0, 4.0) |
